# 60-year-old Female with Edema

**DOI:** 10.5811/cpcem.2022.4.57085

**Published:** 2022-08-08

**Authors:** Nikki A. Cali, Cheyenne Falat, Laura J. Bontempo, J. David Gatz

**Affiliations:** * University of Maryland Medical Center, Department of Emergency Medicine, Baltimore, Maryland; † University of Maryland School of Medicine, Department of Emergency Medicine, Baltimore, Maryland

**Keywords:** emergency medicine, clinicopathological conference, case reports, edema, carcinoid tumor, malignant carcinoid syndrome, carcinoid heart disease

## Abstract

**Introduction:**

Many patients present to the emergency department (ED) with nonspecific, acute-on-chronic complaints. It requires a thorough diagnostic approach and broad differential diagnosis to determine whether there is serious, undiagnosed pathology.

**Case Presentation:**

A 60-year-old female presented to the ED for gradually worsening bilateral lower extremity swelling with associated abdominal distension, ascites, diarrhea, vomiting, and weight loss.

**Discussion:**

This case takes the reader through the evaluation of a patient with acute-on-chronic complaints who presented in a decompensated state.

## CASE PRESENTATION (Dr, Cali**)**

A 60-year-old female presented to the emergency department (ED) with a chief complaint of bilateral lower extremity swelling. She first noticed the swelling three months earlier and felt it was gradually worsening. The swelling initially started in her legs and abdomen but then progressed to her face. She denied any associated pain in her legs but reported that they felt heavy. She denied associated orthopnea, cough, or shortness of breath. She denied any change in activity tolerance but did report a decline in her daily activities due to social distancing because of coronavirus 2019. She also described chronic, brown loose stools over the past year, which were unrelated to her diet. She had loose stools daily, which were not particularly malodorous and not associated with abdominal pain. She also noted non-bloody, non-bilious emesis intermittently over the prior year without any clear, identifiable triggers. She had a remote history of vomiting in the past with panic attacks; so she attributed her vomiting to anxiety. She also noted a 15-pound weight loss during the preceding year, which she attributed to not eating regular meals throughout the day coupled with her persistent vomiting. She denied fevers, chills, night sweats, or chest pain.

Her past medical history included a recent diagnosis of a heart murmur one year earlier. She also had a history of anxiety, panic attacks, depression, an eating disorder (low-baseline caloric intake), and psoriasis. She was previously on fluoxetine and hydroxyzine but took herself off several years earlier as she felt they were not working. She was no longer taking any medication at the time of presentation. She was post-menopausal and had irregular periods prior to menopause. She had no children and had never been pregnant.

**Image 1:**
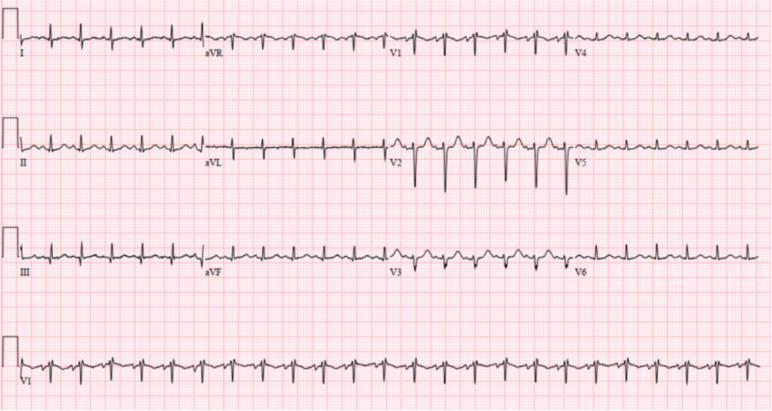
Electrocardiogram of a 60-year-old female with bilateral lower extremity swelling showing sinus tachycardia, normal axis, normal intervals, and no ST or T wave changes. *K*, thousand; *mcL*, microliter; *g*, gram; *dL*, deciliter; mmol, millimole; *L*, liter; *mg*, milligram; *u*, microgram; *pg*, picogram; *ml*, milliliter; *ng*, nanogram.

**Image 2:**
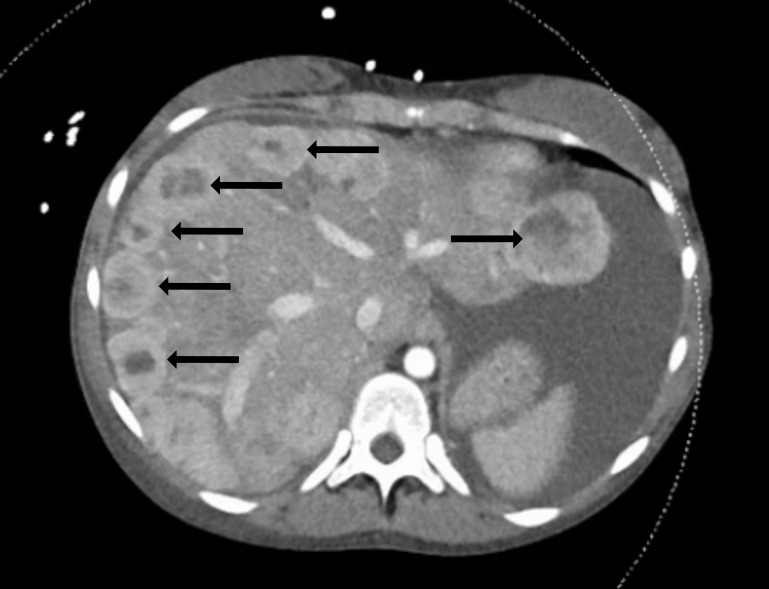
Computed tomography axial view with intravenous contrast of the abdomen of a 60-year-old female with bilateral lower extremity swelling. Multiple hepatic lesions are demonstrated (white arrows).

Vital signs were as follows: temperature 38.4° Celsius, heart rate 140 beats per minute (bpm), blood pressure 120/80 millimeters of mercury, respiratory rate 27 breaths per minute (rpm) and room air oxygen saturation 100%. Her body mass index was 27. Her physical exam was notable for a well-developed female who appeared tired and uncomfortable. Her head, eyes, ear, nose, and throat exam was significant for facial swelling and pupils that were midrange, equal, round, and reactive to light bilaterally. She had moist mucous membranes and no lymphadenopathy or palpable masses. On cardiac exam she had a harsh 4/6 blowing systolic murmur that was loudest at the left sternal border but also auscultated through her back. She was tachypneic but had clear lung sounds. Her abdomen was distended with a fluid wave and dullness to percussion but was nontender. Her extremities were notable for 3+ pitting edema from her feet to her bilateral upper thighs. No upper extremity edema was present. On neurologic exam she had no focal deficits. She was awake, alert, and oriented to person, place, and time. Her skin was warm and dry. Her initial laboratory results (Table 1) showed multiple abnormalities. An electrocardiogram (ECG) was performed (Image 1). A computed tomography (CT) of her abdomen and pelvis with intravenous (IV) contrast was also obtained (Image 2).

The patient was initially treated with IV fluids and piperacillin-tazobactam due to concerns for sepsis with her fever and tachycardia. She acutely worsened after administration of fluids. She subsequently was placed on non-invasive ventilation and was administered IV furosemide. A test was then ordered, and a diagnosis was made.

## CASE DISCUSSION (Dr. Falat)

A chief complaint of “edema” was rarely one that excited me. When I see a patient with edema, it is generally a problem that they have presented with previously, or it is not a terribly difficult diagnostic dilemma. So, when I was handed this case of a 60-year-old female presenting with three months of gradually worsening bilateral lower extremity edema progressing to the abdomen, I will admit that my adrenaline did not immediately surge. However, a few additional historical points did stand out to me that were somewhat atypical: she had no complaints of pain, and she was no longer able to eat regular meals. This was not going to be a simple or typical case of edema.

I like to think of edema in dichotomies: unilateral vs bilateral, and acute vs chronic. The causes of each of these categories vary and are listed in Table 2. This patient presented with chronic bilateral edema, and because my involvement in her case was through our clinicopathological case conference, I immediately felt confident excluding venous insufficiency and lymphedema, as I anticipated there to be a much more interesting etiology of her presentation (being common presentations of common diseases).

Her review of systems was significant for fevers, 15-pound weight loss, facial swelling, bilateral lower extremity edema, abdominal distention, loose brown diarrhea, random episodes of emesis without an obvious trigger, right shoulder pain, psoriatic rash, and nervousness. Could her right shoulder pain or vomiting have been due to atypical anginal symptoms? Could her fever have been indicative of endocarditis? Could her abdominal distention have been due to ascites from liver disease? My mind immediately began elevating cardiac and hepatic diseases in my differential diagnosis.

Her review of systems was also notably negative for shortness of breath, orthopnea, dyspnea on exertion, cough, chest pain, palpitations, abdominal pain, malodorous stool, or change to exercise tolerance. With that, cardiac and pulmonary disease were slightly lowered on my differential diagnosis. While going over her past medical history, medications, surgical history, social history, and family history, I noted her “heart murmur,” eating disorder, psoriasis, and absence of IV drug use. But none of those pieces of information were a game-changer at this point in her puzzle.

Next came her exam. She was tachycardic with a heart rate of 140 bpm, tachypneic with a respiratory rate of 27 rpm, and febrile with a temperature of 38.4°C. She appeared tired and uncomfortable, with a round and swollen face. She had a grade 4/6 blowing systolic murmur that was best heard at the left sternal border and back. She had abdominal distention with findings consistent with ascites but no abdominal tenderness, and she had pitting edema of bilateral lower extremities extending up to her thighs. I found the detailed description of her murmur very interesting; so her cardiac exam anchored my focus.

Cardiac murmurs can also be thought of in dichotomies: systolic vs diastolic and left vs right. Left-heart systolic murmurs are those of aortic stenosis or mitral regurgitation; right-heart systolic murmurs are those of pulmonic stenosis or tricuspid regurgitation; left-heart diastolic murmurs are those of aortic regurgitation or mitral stenosis; and right-heart diastolic murmurs are those of pulmonic regurgitation or tricuspid stenosis. Because hers was a systolic murmur, I reviewed the classic descriptions of those murmurs in further detail.

An aortic stenosis murmur is classically described as “a crescendo-decrescendo systolic murmur along the left sternal border that radiates to the upper right sternal border and into the carotid arteries.”^1^ A mitral regurgitation murmur is classically described as “holosystolic, radiating into the axilla” and “usually heard best at the apex.”^1^ A pulmonic stenosis murmur is classically described as “midsystolic…crescendo-decrescendo” with a “pulmonic ejection click” at the “second intercostal space at the left sternal border.”^2^ I was getting a little overwhelmed with all these descriptions that did not quite fit the murmur of this patient when I read the classic description of a tricuspid regurgitation murmur: a “blowing, holosystolic… murmur best heard at the lower left sternal border.”^2^ Jackpot. This exactly matched the description of the patient’s murmur.

My focus darted to the differential diagnosis for causes of tricuspid regurgitation. The patient had no history of pacemaker placement, so I knew it could not be pacemaker lead trauma. Similarly, I felt confident excluding deceleration injury, sequela of an Ebstein anomaly, or drug-induced disease given there was no history to support any of those entities. With rheumatic heart disease, infective endocarditis, pulmonary hypertension, carcinoid syndrome, ischemic heart disease, myxomatous degeneration, and connective tissue disorder still on the list, I turned to her labs.

This patient had notable abnormalities of leukocytosis, thrombocytosis, and an elevated C-reactive protein, but these are all nonspecific elevations and did not hint toward a diagnosis. Her elevated brain natriuretic peptide indicated perhaps cardiomegaly or strain. Her elevated lactate dehydrogenase and uric acid revealed the possibility of increased cell turnover. And her elevated ammonia level suggested the possibility of primary hepatic failure or the presence of a portosystemic shunt such as in the setting of portal venous hypertension secondary to vascular congestion. I interpreted her hypokalemia as confirming her reports of vomiting and diarrhea. Additionally, decreased protein and albumin, such as hers, can be found in cases of nephrotic syndrome but also in cases of hepatic disease, causing increased capillary permeability and decreased albumin synthesis.

Along with her lab abnormalities, she had notable normal values of magnesium (which was important to check given her hypokalemia), blood urea nitrogen and creatinine (which is important since I was considering renal disease), aminotransferases (which is important since I was considering hepatic disease), thyroid-stimulating hormone (which was important to check given her profound tachycardia), and troponin (which was important to check given her worsened vs new cardiac murmur).

After reviewing her labs, I significantly lowered primary hepatic failure and renal failure on my differential diagnosis because otherwise I would have expected more profound derangements in her labs. And after reviewing her ECG, which showed no right axis deviation, and her chest radiograph (CXR), which did not reveal increased pulmonary vascular markings, I lowered and ultimately removed pulmonary hypertension and pulmonary disease off my differential.

This left me with cardiac disease as the cause of her edema, and specifically cardiac valvular disease since her exam perfectly described tricuspid regurgitation. I knew that an echocardiogram was at some point going to be performed, which, I presumed, would confirm the diagnosis of tricuspid regurgitation. But tricuspid regurgitation alone did not explain all her associated symptoms on her review of systems. And I was still left with the nagging question of “Why?” Why had she developed tricuspid regurgitation?

Her negative troponin, absence of chest pain, and absence of Q waves or ischemic changes on her ECG allowed me to cross off ischemic heart disease. I removed myxomatous degeneration and connective tissue disorders off the differential diagnosis of tricuspid regurgitation because her history did not reveal any other findings to anchor these diagnoses. Her absence of reported IV drug use, human immunodeficiency virus, prosthetic heart valves, dental work, or rheumatic heart disease allowed me to remove both infective endocarditis and rheumatic heart disease. Which left me with carcinoid syndrome.

Never having previously diagnosed a new case of carcinoid syndrome in my career, I needed to review this entity to make sure I was not way off base. Carcinoid syndrome is a constellation of symptoms arising from secretion of substances from a variety of neuroendocrine tumors (NET), with presentations varying based on the location of the primary tumors and the substances they secrete. The tumors tend to be indolent, but metastases (liver, lymph nodes, peritoneum) are common, and carcinoid syndrome usually presents once hepatic metastases arise. And after staring at the CT of her abdomen, I noticed her clear hepatic metastases staring back at me. Furthermore, carcinoid tumors are associated with cardiac fibrosis (thought to be caused by serotonin secretion from the tumors) of the right-sided valves resulting in tricuspid regurgitation and pulmonic stenosis.

Equipped with this knowledge, never had I felt so confident in making a diagnosis that I had never previously made. The patient’s symptoms and the pathophysiology of carcinoid syndrome came together like a perfect symphony. Her fevers were in the setting of flushing vs neoplastic symptoms. Her edema was due to tricuspid regurgitation causing congestive hepatopathy vs hepatic metastases. Her diarrhea and tachycardia were from her carcinoid secretory effects. Her weight loss, abdominal distention, and irregular meal habits were due to early satiety from hepatomegaly and ascites. Her labs indicated increased neoplastic cell turnover. And her beautifully described tricuspid regurgitation murmur clinched the diagnosis since right-sided valvular cardiac fibrosis is associated with carcinoid syndrome. The most common way to diagnosis carcinoid syndrome is by a 24-hour urine collection of 5-hydroxyindoleacetic acid (5-HIAA). And with that test, I’ll never look at edema with boredom again.

## CASE OUTCOME (Dr. Cali)

After the CT of the abdomen and pelvis showed multiple enhancing nodules scattered throughout the liver, the leading diagnosis was metastatic lesions, but from an unknown primary source. The patient presented to the ED in decompensated right heart failure with signs of volume overload on clinical exam including pitting edema and abdominal ascites. Her 4/6 blowing holosystolic murmur, which was best auscultated overlying the left upper sternal border, was of significant concern, and an echocardiogram was obtained. The echocardiogram showed moderate to severe tricuspid valve insufficiency, mild to moderate pulmonary valve stenosis, and mildly depressed right ventricular systolic function. In conjunction with the patient’s liver lesions, these findings on echocardiogram led to a strong suspicion for carcinoid heart disease.

To confirm the diagnosis of carcinoid heart disease, the liver tumors were biopsied. Immunostains of the biopsies were positive for chromogranin and synaptophysin, negative for cytokeratin 7, cytokeratin 20, anti-hepatocyte specific antigen 1, and beta-catenin, which was consistent with a well-differentiated NET.

The patient was started on medical management including the somatostatin analog octreotide to help with her diarrhea. She ultimately required bivalvular replacement of both the tricuspid and pulmonic valves due to the extent of her cardiac disease. Her hospital course was complicated by multiple infections including endocarditis, and she ultimately did not survive the disease, dying less than two months after her initial ED presentation.

## RESIDENT DISCUSSION

Carcinoid tumors are extremely rare NETs that occur in approximately 1 in 100,000 individuals in the general population.^3^ They are commonly found in the gastrointestinal system, anywhere from the embryologic foregut to the hindgut, with the appendix and terminal ileum being the most common sites.^4^ Other less common sites for carcinoid tumors include the respiratory and genitourinary tracts.

Carcinoid tumors are indolent growing tumors with a peak incidence occurring between the sixth and seventh decade of life.^5^ These tumors remain asymptomatic for several years and do not produce symptoms until they metastasize, which makes an early diagnosis challenging. Once carcinoid tumors metastasize, they have the potential to produce carcinoid syndrome, which is characterized by intermittent facial flushing, intractable secretory diarrhea, and bronchoconstriction.^4^ However, classic carcinoid syndrome occurs in fewer than 10% of patients with carcinoid tumors; so, a high index of clinical suspicion is imperative to make the diagnosis.^6^

Carcinoid syndrome is caused by vasoactive substances, such as serotonin, that are released by the tumor into the blood stream and evade hepatic degradation. When carcinoid tumors exist in the gastrointestinal system in isolation, serotonin and other vasoactive substances are degraded by monoamine oxidases in the liver, lungs, and brain into secretory byproducts, most notably 5-HIAA, which do not manifest into clinical symptoms.^7^ When carcinoid tumors metastasize to the liver they evade hepatic degradation and their vasoactive substances are released into systemic circulation to exert downstream effects on the right side of the heart causing carcinoid heart disease.

Carcinoid heart disease was first reported in 1954. Although the mechanism behind its development is not fully understood, serotonin is considered a major initiator of the fibrotic process.^3^,^6^ When the right side of the heart is exposed to serotonin from systemic circulation, this results in endocardial damage causing thickening, retraction, and fixation of the right heart valves, valvular dysfunction, and ultimately right heart failure.^8^ Pathognomonic echocardiographic features include immobility of valve leaflets resulting in tricuspid valve regurgitation and pulmonary stenosis.^4^ The right side of the heart is predominately affected because serotonin is degraded in the pulmonary circulation before it can reach the left side of the heart in its active form.

The diagnosis of carcinoid syndrome and carcinoid heart disease is multifactorial. First, the astute clinician must have a high index of suspicion based on the patient’s history and physical exam. History may include episodic flushing episodes, which can be triggered by alcohol, exercise, or tyramine-containing foods in addition to complaints of diarrhea and wheezing. The patient should also be screened for any murmurs on cardiac exam.^7^ In the setting of advanced carcinoid heart disease, the patient will present with signs of right-sided heart failure including jugular venous distension, ascites, dependent edema, weight gain, and hepatomegaly. Brain natriuretic peptide will likely be elevated, but this is neither sensitive nor specific to carcinoid heart disease. Key diagnostic testing includes a 24-hour urine 5-HIAA sample, which has a sensitivity of 73% and specificity of 100% for diagnosing carcinoid.^7^

In a patient who presents with a heart murmur and signs of heart failure, an echocardiogram is imperative to diagnosing carcinoid heart disease. Imaging such as CXRs and ECGs are typically nonspecific but should be included in the patient’s workup. Computed tomography is futile in detecting primary carcinoid tumors but is helpful to evaluate extent of tumor spread.^7^ Unfortunately, because many patients present later in their disease course, CTs are often the first identification of metastatic spread. A more sensitive imaging modality to detect smaller carcinoid tumors is somatostatin receptor scintigraphy, a radiolabeled imaging test based on the principle that approximately 88% of carcinoid tumors possess somatostatin receptors. 9 This helps in diagnosing earlier, primary tumors that CT can miss.

Treatment of carcinoid disease requires a multidisciplinary approach with surgical and medical oncologists, as well as gastroenterologists. In early, non-metastatic disease, surgical resection can be curative.^4^ In the presence of carcinoid syndrome, medical management consists of somatostatin analogs such as octreotide and lanreotide to control symptoms of flushing and diarrhea caused by secretion of tumor bioactive agents.^6^,^10^ However, symptom control is typically temporary, and tumor debulking including liver resection is considered in more severe cases, when possible. In the presence of cardiac involvement with valvular dysfunction, additional management is focused on treating right-sided heart failure. Cardiac valve surgery is proposed as a final curative intervention only for patients who are mildly symptomatic due to increased perioperative mortality with more advanced heart failure.^6^ Once cardiac involvement is present, it cannot be reversed without surgical intervention, which is why early detection and intervention of carcinoid tumors remains a strong prognostic indicator of morbidity and mortality.

**KEY TEACHING POINTS**

1. If you lack clinical suspicion for carcinoid syndrome, you will never make the diagnosis.
2. Carcinoid syndrome should be considered in any patient with persistent, unexplained diarrhea or a new right-sided heart murmur without an alternate explanation.
3. Carcinoid tumors typically become symptomatic after metastasizing to the liver.
4. Tricuspid regurgitation and pulmonary stenosis are hallmark echocardiogram findings in carcinoid heart disease.

## Figures and Tables

**Table 1:** Laboratory results of a 60-year-old female with bilateral lower extremity swelling.

Blood test	Patient value	Normal range
Complete blood count		
White blood cells	16.1 K/mcL	4.5 – 13.0 K/mcL
Hemoglobin	9.2 g/dL	12 – 16 g/dL
Hematocrit	29.3%	36.0 – 46.0%
Platelets	578 K/mcL	153 – 367 K/mcL
Differential		
Polymorphonuclear leukocytes	78.5%	42.6–74.5%
Lymphocytes	13.9%	20.8–50.5%
Monocytes	6.1%	2–10%
Eosinophils	0.2%	1–3%
Serum chemistries		
Sodium	137 mmol/L	136–145 mmol/L
Potassium	2.7 mmol/L	3.5–5.1 mmol/L
Chloride	109 mmol/L	98–107 mmol/L
Bicarbonate	17 mmol/L	21–30 mmol/L
Blood urea nitrogen	15 mg/dL	7–17 mg/dL
Creatinine	0.66 mg/dL	0.42–0.92 mg/dL
Glucose	136 mg/dL	70–99 mg/dL
Magnesium	1.9 mg/dL	1.6–2.6 mg/dL
Phosphorus	2.6 mg/dL	2.8–4.6 mg/dL
Total protein	5.2 g/dL	6.3–8.6 g/dL
Albumin	2.3 g/dL	3.5–5.2 g/dL
Hepatic Studies		
Total bilirubin	1.0 mg/dL	0.3–1.2 mg/dL
Aspartate aminotransferase	37 u/L	14–36 u/L
Alanine aminotransferase	20 u/L	0–34 u/L
Alkaline phosphatase	153 u/L	50–130 u/L
Cardiac Studies		
N-terminal prohormone of brain natriuretic peptide	2,200 pg/mL	<900 pg/mL
Troponin	<0.02 ng/mL	<0.06 ng/mL
Coagulation studies		
Prothrombin time	16.5 seconds	12.1–15.0 seconds
Partial thromboplastin time	36 seconds	25–38 seconds
International normalized ratio	1.3	
Other		
C-reactive protein	4.3 mg/dL	<1.0 mg/dL
Lactate dehydrogenase	696 units/L	240–670 units/L
Uric acid	8.0 mg/dL	2.6–6.0 mg/dL
Ammonia	60 mcmol/L	9–30 mcmol/L
Thyroid stimulating hormone	1.88 mIU/L	0.50–4.50 mIU/L
Respiratory viral panel		
SARS-CoV-2 (COVID-19) RNA	Not detected	
Influenza A RNA amplification	Not detected	
Influenza B RNA amplification	Not detected	
Parainfluenza 1,2,3,4 Virus RNA amplification	Not detected	
Rhinovirus/enterovirus RNA amplification	Not detected	
RSV RNA amplification	Not detected	

*dL*, deciliter; *L*, liter; *mg*, milligram; *mcmol*, micromoles; *mIU*, milli-international units; *SARS-CoV-2*, severe acute respiratory syndrome coronavirus 2; *COVID-19*, coronavirus 2019; *RNA*, ribonucleic acid; *RSV*, respiratory syncytial virus.

**Table 2:** Differential diagnosis of lower extremity edema.

	Acute	Chronic
Unilateral	Deep vein thrombosis Cellulitis Compartment syndrome Muscle rupture	Venous insufficiency Lymphedema Malignancy Complex regional pain syndrome
Bilateral	Medications (calcium channel blockers, steroids, hormones) Bilateral or pelvic deep vein thromboses	Venous insufficiency Lymphedema Systemic (cardiac, hepatic, renal, or pulmonary) disease
